# Optimizing Final Pathology Determination in Endometrial Cancer: The Role of PET/CT, MRI, and Biopsy in Serous, Mixed Cell, Clear Cell, and Grade 3 Endometrioid Subtypes

**DOI:** 10.3390/diagnostics15060731

**Published:** 2025-03-14

**Authors:** Gözde Şahin, Ayşe HazırBulan, Işık Sözen, Nilüfer Çetinkaya Kocadal, İsmet Alkış, Aytül Hande Yardımcı, Burcu Esen Akkaş, Hilal Serap Arslan

**Affiliations:** 1 Gynecologic Oncology Clinic, Basakşehir Cam and Sakura City Hospital, Istanbul 34480, Turkey; aysehazirbulan@gmail.com (A.H.); isiksozen@gmail.com (I.S.); cetinkayanilufer@gmail.com (N.Ç.K.); ismetalkis@hotmail.com (İ.A.); 2 Radiology Clinic, Basakşehir Cam and Sakura City Hospital, Istanbul 34480, Turkey; yahandeoo@yahoo.com; 3 Nucleer Medicine Clinic, Basakşehir Cam and Sakura City Hospitali, Istanbul 34480, Turkey; burcuesen.akkas@sbu.edu.tr; 4 Patology Clinic, Basakşehir Cam and Sakura City Hospital, Istanbul 34480, Turkey; dr.seraparslan@gmail.com

**Keywords:** endometrial cancer, endometrial biopsy, MRI, PET/CT, final pathology

## Abstract

**Background:** Accurate and timely diagnosis of endometrial cancer is crucial for guiding effective treatment and improving patient survival. Endometrial cancer diagnosis, staging, metastasis detection, and treatment planning utilize endometrial biopsy, magnetic resonance imaging (MRI), and positron emission tomography-computed tomography (PET/CT) scanning as crucial diagnostic modalities. Aggressive subtypes such as serous, mixed cell, clear cell, and grade 3 endometrioid carcinomas present considerable diagnostic and therapeutic obstacles given their unfavorable prognosis, underscoring the importance of accurate preoperative evaluation. **Methods:** A retrospective analysis was conducted using data from seventy patients diagnosed with serous, mixed cell, clear cell, or grade 3 endometrioid endometrial cancer, who received surgical treatment from 2020 to 2023. To assess the diagnostic capabilities of each modality in determining final pathology and disease staging, a comparison was performed using results from preoperative endometrial biopsy, MRI, PET/CT, and postoperative histopathology. Cohen’s kappa coefficient was employed to determine the level of agreement observed between pre- and postoperative results. **Results:** Endometrial biopsy demonstrated moderate yet statistically significant concordance with definitive histopathological diagnoses (κ = 0.537, *p* < 0.001); however, diagnostic errors were observed, especially in instances of mixed and clear cell carcinomas. MRI demonstrated efficacy in identifying local tumor invasion, yet its capacity to detect distant metastases was demonstrably limited. PET/CT was most effective in identifying distant metastases and omental involvement in advanced-stage disease. **Conclusions:** Definitive pathological diagnosis and staging of endometrial carcinoma are effectively established using endometrial biopsy and MRI. The utility of PET/CT is particularly pronounced in identifying distant metastases in patients with serous carcinoma and advanced-stage disease. Integrating biopsy, MRI, and PET/CT into a multimodal diagnostic strategy enhances diagnostic accuracy and enables personalized treatment planning, particularly for aggressive tumor subtypes.

## 1. Introduction

Among gynecological malignancies, endometrial cancer demonstrates the highest incidence, typically associated with a positive prognostic outlook. A challenging clinical subgroup, representing 10–20% of cases, exhibits aggressive behavior, higher recurrence, and reduced survival. Endometrioid, serous, clear cell, and mixed cell carcinomas of grade 3 are included in this subgroup [1,2].

A substantially worse prognosis is associated with aggressive histological subtypes of endometrial carcinoma relative to low-grade endometrioid cancers [3]. Clear cell and serous carcinomas demonstrate a statistically significant increased likelihood of distant metastasis. Early detection of these subtypes allows clinicians to personalize treatment protocols, frequently necessitating extensive surgical procedures, chemotherapy, and/or radiotherapy [4]. Optimal patient outcomes necessitate accurate diagnostic procedures and staging.

An endometrial biopsy serves as the primary diagnostic step in assessing endometrial cancer, providing an initial determination of histological subtype and grade [5,6]. Despite its high effectiveness in identifying endometrioid cancers, the test’s accuracy is limited when identifying non-endometrioid and high-grade subtypes [7]. This limitation underscores the need for incorporating additional imaging modalities to achieve greater diagnostic accuracy.

Magnetic resonance imaging (MRI) is crucial for preoperative assessment, providing detailed visualization of myometrial invasion, cervical stromal involvement, and lymph node metastasis, which is essential for precise cancer staging. The application of advanced MRI techniques, specifically diffusion-weighted imaging (DWI) and apparent diffusion coefficient (ADC) analysis, facilitates improved detection of tumor grade and lymphovascular space invasion (LVSI) [8,9]. Nevertheless, preoperative MRI results do not consistently correlate with definitive pathology, thus necessitating supplementary diagnostic methods.

Positron emission tomography-computed tomography (PET/CT) plays a crucial role in detecting lymph node involvement and distant metastases. This method shows high sensitivity to metastatic disease, yet its ability to detect local tumor invasion, myometrial invasion, and early-stage disease is compromised. The value of PET/CT is especially pronounced in serous carcinomas, which frequently exhibit distant metastases, and its established role in treatment planning for these cases is well-documented [10,11].

A multimodal approach is crucial given the diagnostic challenges posed by high-risk endometrial cancers. Although endometrial biopsy serves as a primary diagnostic method, its inability to reliably identify high-grade and non-endometrioid cancers necessitates the use of additional imaging techniques. MRI provides enhanced anatomical detail, in contrast to PET/CT, which excels in detecting distant metastases, particularly within aggressive disease variants. This study aims to assess the diagnostic accuracy of endometrial biopsy, MRI, and PET/CT in predicting final pathology in high-risk endometrial cancer subtypes, focusing specifically on the sensitivity and specificity of each modality in identifying key histologic features. This study uses preoperative and final pathological results to suggest a more thorough diagnostic approach for complex cases.

## 2. Materials and Methods

This retrospective study involved patients diagnosed with serous, mixed cell, clear cell, or grade 3 endometrioid endometrial carcinoma who attended the Gynecologic Oncology Surgery Clinic during 2020–2023 and underwent subsequent surgical procedures. Preoperative endometrial biopsy results, imaging studies, and final pathology reports were reviewed for each patient to determine a diagnosis. For each patient, endometrial sampling, MRI, and PET/CT data were obtained, followed by an assessment of concordance between preoperative findings and definitive pathological results. To ensure homogeneity, the study excluded patients with non-endometrial cancers, recurrent endometrial cancers, or other endometrial cancer subtypes. The study received ethical approval from the Başakşehir Çam and Sakura City Hospital Ethics Committee, ensuring compliance with all relevant ethical guidelines.

Distant metastases and tumor metabolic activity were assessed via PET/CT scans utilizing a Philips Ingenuity TF PET/CT system. PET/CT is especially valuable for detecting distant metastases, thus impacting staging and treatment choices. Myometrial invasion and cervical involvement, crucial factors in surgical planning and staging, were evaluated using a 1.5 Tesla Philips Ingenia MRI to assess local tumor characteristics. Endometrial tissue samples were obtained via Pipelle curettage for histopathological examination to establish an initial diagnosis. This initial sampling method, crucial for endometrial cancer diagnosis, reveals key details about the tumor’s grade and its precise histological makeup.

Cervical cytology results were classified into four clinically significant categories: Normal/Benign, Low-Grade Abnormalities, High-Grade Abnormalities, and Malignant Findings. Specific cytological findings included in each category are detailed below.

Statistical analyses were performed using SPSS version 24. The statistical methodology was adapted to accommodate the nature and distribution of the variables. Descriptive statistics were computed for both continuous and categorical variables. For normally distributed data, numerical variables were summarized using the mean and standard deviation; the median, minimum, and maximum values were reported for non-normally distributed data. Frequency and percentage distributions [*n* (%)] of patient and disease characteristics were computed for categorical variables to provide a comprehensive overview. Numerical data were analyzed using independent sample *t*-tests for two-group comparisons (assuming normality) and one-way ANOVAs for comparisons involving three or more groups. Non-parametric analyses were conducted for numerical variables not exhibiting a normal distribution; the Mann–Whitney U test was utilized for two-group comparisons, while the Kruskal–Wallis test was employed for comparisons involving three or more groups. Chi-square analysis determined the presence of associations among the categorical variables. Cohen’s kappa analysis was performed to assess the agreement between preoperative endometrial biopsy results and postoperative histopathological diagnoses, accounting for chance agreement. Kappa values were interpreted based on standard thresholds. A two-tailed test was used for all hypotheses, with statistical significance established at *p* < 0.05.

## 3. Results

The study population consisted of 70 patients exhibiting various endometrial carcinoma histology. The predominant subtype was serous carcinoma (48.6%, *n* = 34), followed by endometrioid adenocarcinoma (40%, *n* = 28), clear cell carcinoma (2.9%, *n* = 2), and mixed cell carcinoma (8.6%, *n* = 6). A mean duration of 24.2 ± 11.24 months post-diagnosis was observed in the cohort. Endometrioid adenocarcinoma patients had the longest time since diagnosis (27.07 ± 12.37 months), in contrast to clear cell carcinoma patients (18 ± 12.73 months). This difference, however, was statistically insignificant (*p* = 0.38). The average patient age was 64 years (±9.57 years). No statistically significant difference (*p* = 0.127) was found in mean age between the serous carcinoma cohort (66.35 ± 9.57 years) and patients with other histological types. Across histological subtypes, mean body weight was 80.79 ± 14.29 kg (*p* = 0.546), and mean height was 159.13 ± 5.35 cm (*p* = 0.719).

Most patients (62.86%) were obese or severely obese, with no significant difference between subgroups (*p* = 0.514) according to BMI classification. Morbid obesity was considerably more common among patients with endometrioid adenocarcinoma (14.29%). Most patients received diagnoses of advanced-stage disease. Stage III was the prevailing stage, making up 32.86% of the total. In contrast, patients with endometrioid adenocarcinoma had a higher occurrence of Stage I (32.14%). Statistical analysis revealed no significant variation in disease stage distribution among subtypes (*p* = 0.321). Mixed cell and serous carcinomas had the highest mortality rates (16.67% and 11.76%, respectively), but this difference was not statistically significant (*p* = 0.828). Table 1 provides a detailed clinical profile of the endometrial carcinoma patient cohort.

### 3.1. Comparison of Preoperative Diagnostic Findings with Postoperative Histological Findings

Preoperative endometrial biopsy results showed significant discrepancies (*p* < 0.001) when compared with postoperative histological diagnoses across the various subtypes. Preoperative biopsies revealed serous carcinoma in 48.57% of instances, exhibiting substantial concordance (93.10%) with postoperative diagnoses of serous carcinoma; however, significant misclassification was observed in endometrioid adenocarcinoma (20%) and mixed carcinoma (12.5%). Preoperative biopsy identified endometrioid adenocarcinoma in 40% of cases, which corresponded with 76% of the postoperative diagnoses. However, 50% of cases initially diagnosed as mixed carcinoma exhibited overlapping histological features with endometrioid adenocarcinoma, highlighting potential classification challenges. These discrepancies were further examined using Cohen’s kappa analysis, which demonstrated moderate agreement (κ = 0.537, SE = 0.074, *p* < 0.001). Findings show moderate consistency between pre- and postoperative evaluations.

Groups showed similar smear results (*p* = 0.64), predominantly benign (67.14%). While serous carcinomas exhibited a higher frequency of high-grade abnormalities, this finding lacked statistical significance (*p* = 0640). Analysis of MRI scans indicated the presence of mass lesions in 38.57% of patients (*p* = 0.587). A significant majority of cases (81.43%) exhibited local involvement across all subtypes; distant metastasis was observed in a small minority (7.14%). Across all groups, PET/CT scans showed a significant increase in involvement, reaching 94.29%. Notably, a significantly higher uptake rate was observed in clear cell and mixed carcinomas (*p* = 0.042).

SUVmax values were measured from the primary tumor. The results indicate no significant difference in SUVmax across histopathological subtypes (*p* = 0.336) or survival status (*p* = 0.376). However, SUVmax was significantly lower in patients with distant metastases compared to those without (*p* = 0.017), suggesting that metabolic activity may vary with disease dissemination. These findings reinforce the potential role of metabolic imaging in assessing disease burden preoperatively (Table 2, Table 3, Table 4 and Table 5).

A mean SUVmax of 17.96 ± 10.18 was observed in endometrioid adenocarcinomas, though this did not reach statistical significance (*p* = 0.336). Table 5 displays the comparative analysis of diagnostic results obtained before and after the operation.

### 3.2. Comparison of Preoperative and Postoperative Findings in Relation to Mortality Outcomes

A comparison was performed to understand the relationship between preoperative and postoperative findings and mortality outcomes. In both groups, preoperative smears showed a predominance of benign cytology (78.18% in the survivor group and 80% in the deceased group; *p* = 0.719). Although a greater frequency of high-grade abnormalities was observed in the deceased cohort (20%) compared to the surviving cohort (10.91%), this disparity lacked statistical significance.

Analysis of preoperative endometrial biopsies indicated serous carcinoma in 57.14% of deceased patients and 47.62% of surviving patients. Conversely, endometrioid adenocarcinoma was more prevalent among survivors (41.27%) than deceased patients (28.57%) (*p* = 0.673). Consistent postoperative histopathological diagnoses were observed across groups, with serous carcinoma representing the most frequent subtype in both (deceased: 42.86%; survivors: 41.27%; *p* > 0.999).

A statistically non-significant difference (*p* = 0.417) in the frequency of mass lesions was observed between deceased (57.14%) and surviving (36.51%) patients on MRI. Analysis of PET/CT scans revealed widespread disease involvement exceeding 93% in both groups, showing no significant difference in SUVmax (*p* = 0.376). Analysis revealed no significant variation in any parameter between the survivor and deceased patient cohorts (Table 6).

### 3.3. Comparison of Clinical, Radiological, and Pathological Findings Across Cancer Stages

Across all stages, the smear results showed a predominance of benign findings, with Stage II recording the highest rate (92.86%) and Stage IV the lowest (63.64%) (*p* = 0.434). In Stage IV, high-grade abnormalities occurred with significantly higher frequency (27.27%) than in the earlier stages of the disease. Analysis of preoperative endometrial biopsies revealed serous carcinoma as the predominant diagnosis, particularly in advanced stages (76.92% in Stage IV versus 35.29% in Stage I), whereas endometrioid adenocarcinoma demonstrated a higher incidence in early-stage disease (*p* = 0.321). Postoperative histopathological examination demonstrated that serous carcinoma was considerably more frequent in Stage IV (76.92%) than in Stage I (23.53%), while endometrioid adenocarcinoma exhibited a higher frequency in the earlier stages (*p* = 0.164).

MRI results demonstrated similar patterns of mass lesions and local involvement among all stages; nevertheless, the incidence of distant metastasis was considerably higher in Stage IV (23.08%) compared to other stages (*p* = 0.053). Analysis of PET/CT scans demonstrated substantially elevated occurrences of omental involvement and distant metastasis in Stage IV patients (*p* < 0.001). No significant differences were observed in SUVmax values across stages (*p* = 0.349). Table 7 compares clinical, radiological, and pathological findings across cancer stages, highlighting the higher rates of serous carcinoma and metastasis in Stage IV.

A statistically significant difference was observed between smoking status and histopathological subtypes (*p* = 0.019) (Table 8).

## 4. Discussion

The incidence of endometrial cancer, the most common gynecological malignancy worldwide, is steadily increasing. This trend is particularly evident in developed countries and is associated with factors such as rising obesity rates and longer life expectancy [12]. While most endometrial cancers are detected at an early stage and have a favorable prognosis, high-grade aggressive subtypes such as serous, clear cell, mixed cell carcinomas, and grade 3 endometrioid adenocarcinomas are linked to poorer clinical outcomes [5]. Given the disproportionate impact of these aggressive variants on disease recurrence and mortality, accurate preoperative assessment is crucial for determining the most effective treatment strategy [5,13].

This study highlights the importance of integrating preoperative biopsy, MRI, and PET/CT for the accurate diagnosis and staging of endometrial carcinoma. Endometrial biopsy remains the gold standard for initial diagnosis, as it effectively identifies malignant processes and distinguishes between histological subtypes [14]. However, our findings indicate only a moderate concordance between preoperative biopsy and postoperative histopathology (κ = 0.537, *p* < 0.001). This result aligns with previous studies demonstrating the limitations of biopsy in accurately classifying aggressive and mixed histological subtypes [6,7]. Notably, misclassification was more prevalent in mixed and clear cell carcinomas, emphasizing the need for improved sampling techniques or supplementary molecular diagnostics to enhance diagnostic accuracy [15,16]. Postoperative adjuvant therapy decisions are typically based on surgical specimen pathology worldwide. While preoperative biopsy serves as a crucial guide for patients requiring neoadjuvant therapy, it is essential to recognize that surgical specimen pathology remains the most reliable source for determining the need for adjuvant therapy. In our study, adjuvant treatment planning was based on surgical specimen pathology results and was administered accordingly [17].

Lin et al. emphasized the necessity of a multimodal imaging approach in the preoperative evaluation of endometrial carcinoma [18]. MRI remains the preferred imaging modality for assessing local tumor characteristics such as myometrial invasion and cervical involvement, which are critical for surgical planning [19,20]. However, its limitations in detecting distant metastases underscore the complementary role of PET/CT. PET/CT has demonstrated superior sensitivity in identifying para-aortic and distant metastatic lymph nodes, particularly in cases of serous carcinoma, which is associated with a more aggressive clinical course [21,22]. Nevertheless, the reduced sensitivity of PET/CT in detecting small metastatic foci and early-stage disease must be considered in the integration of imaging modalities into clinical decision-making [22,23].

Our findings emphasize the importance of MRI and PET/CT in the preoperative evaluation of endometrial cancer, while the literature also highlights the role of CT in assessing advanced-stage disease and metastatic spread. The 2023 FIGO updates have further underscored the significance of CT in identifying peritoneal metastasis [2,24,25,26]. A study by Mazzei et al. reported high sensitivity and specificity of CT in detecting peritoneal carcinomatosis [27]. However, due to its lower soft-tissue contrast, CT is considered less effective than MRI in assessing myometrial invasion and local staging. In our study, MRI was found to be crucial in evaluating the depth of myometrial invasion and guiding surgical planning, while PET/CT proved to be particularly valuable in assessing lymph node metastases and distant spread in aggressive subtypes such as serous carcinoma. CT remains a widely available imaging modality, yet its reliance on size criteria limits its accuracy in evaluating distant metastases and lymph node involvement [28,29]. Nevertheless, in the absence of PET/CT, CT can serve as a valuable tool for the general assessment of pelvic and abdominal regions, particularly in detecting enlarged nodules and gross soft tissue masses in advanced-stage disease.

Compared with the existing literature, our study supports the use of multimodal imaging approaches to enhance diagnostic accuracy, particularly in aggressive endometrial cancer subtypes. Given the observed discrepancy between preoperative biopsy and final pathology results, a more integrated diagnostic approach that includes molecular profiling, alongside imaging modalities, may improve risk stratification and aid in more precise adjuvant therapy decision-making [30,31].

MRI remains the optimal imaging technique for local staging, demonstrating high accuracy in assessing the depth of myometrial invasion, cervical stromal involvement, and lymph node metastases [32,33]. Our study found that while MRI exhibited high sensitivity in detecting local tumor spread, it was less effective in identifying distant metastases, consistent with previous research [33,34,35]. The accurate measurement of myometrial invasion depth is of great importance in surgical planning and determining the necessity of lymphadenectomy [36]. Our findings reinforce the value of MRI in preoperative planning, as deeper myometrial invasion was more frequently observed in patients with advanced-stage disease.

Recent MRI techniques offer significant advancements in the assessment of endometrial cancer. Diffusion-weighted imaging (DWI) has emerged as a crucial tool for evaluating tumor cellularity and myometrial invasion depth [37].

Additionally, PET/CT has proven to be highly effective in detecting lymph node metastases and distant disease, particularly in serous carcinomas, which have a high propensity for metastasis [38,39]. The strong correlation between PET/CT imaging data and surgical pathology in serous carcinoma supports its role in staging and guiding adjuvant therapy. However, PET/CT remains limited in its ability to detect small-volume disease and early metastases, as corroborated by previous research [40,41]. Despite these limitations, PET/CT surpasses other modalities in identifying para-aortic lymph node involvement and assessing treatment response [42].

A comprehensive preoperative evaluation requires the synthesis of findings from biopsy, MRI, and PET/CT. A multimodal diagnostic approach has been shown to improve staging accuracy and enable personalized treatment plans that reduce the risk of recurrence and enhance survival outcomes. This approach is particularly critical in high-risk subtypes such as serous carcinoma, where early detection and aggressive surgical and adjuvant therapies are essential for improved prognosis [43,44].

Among the seven deceased patients analyzed in our study, most were over the age of 70 and diagnosed with either serous carcinoma (42.9%) or endometrioid adenocarcinoma (42.9%). This finding aligns with the well-established aggressive nature of serous carcinoma, which is associated with poor prognosis even in early-stage presentations [45]. The increased rates of distant metastasis and omental involvement in advanced disease in our study population further reinforce the prognostic value of accurate staging.

Future research should prioritize the development of improved diagnostic tools for rare histological subtypes, incorporating molecular profiling to facilitate more precise risk stratification and individualized treatment protocols. Additionally, the advancement of targeted therapies and immunotherapies holds significant potential for improving outcomes in aggressive endometrial cancers.

The findings of this study highlight the necessity of a combined diagnostic strategy incorporating biopsy, MRI, and PET/CT imaging in endometrial carcinoma, leveraging the strengths of each modality to improve diagnostic precision, guide personalized treatment planning, and ultimately enhance patient survival and overall health outcomes.

## 5. Conclusions

This study evaluated the diagnostic performance of biopsy, MRI, and PET/CT in the detection and staging of endometrial carcinoma. Although preoperative biopsies showed a degree of concordance with postoperative histopathology, especially in serous carcinomas, considerable disparities were identified among endometrioid and mixed carcinoma diagnoses. MRI effectively demonstrated local disease involvement; however, its sensitivity in detecting distant metastases was inferior to that of PET/CT, particularly in advanced-stage and serous carcinomas. PET/CT imaging revealed significantly greater tracer uptake in clear cell and mixed carcinomas. The present findings highlight the necessity of a multimodal diagnostic strategy, integrating biopsy, MRI, and PET/CT, to improve diagnostic precision and facilitate personalized treatment plans. Additional studies are necessary to understand the diagnostic accuracy, especially concerning the identification of less prevalent histological subtypes and early metastatic dissemination.

## Figures and Tables

**Table 1:** Clinical and Demographic Characteristics of Patients by Endometrial Carcinoma Subtypes.

	Overall (*n* = 70)	Serous Carcinoma (*n* = 34)	Endometrioid Adenocarcinoma (*n* = 28)	Clear Cell Carcinoma (*n* = 2)	Mixed Cell Carcinoma (*n* = 6)	*p*-Value
Age (years)	64 ± 9.76	66.35 ± 9.57	60.68 ± 9.48	68.5 ± 9.19	64.67 ± 10.03	0.127
Weight (kg)	80.79 ± 14.29	79.24 ± 10.86	83.71 ± 18.09	85 ± 7.07	74.5 ± 12.11	0.546
Height (cm)	159.13 ± 5.35	159.38 ± 5.13	159.21 ± 6.01	160 ± 0	157 ± 4.47	0.719
Months Since Diagnosis	24.2 ± 11.24	22.56 ± 9.76	27.07 ± 12.37	18 ± 12.73	22.17 ± 13.06	0.380
Body Mass Index (BMI)						0.514
*Normal*	5 (7.14%)	1 (2.94%)	3 (10.71%)	0 (0.00%)	1 (16.67%)	
*Overweight*	15 (21.43%)	11 (32.35%)	3 (10.71%)	0 (0.00%)	1 (16.67%)	
*Obese*	36 (51.43%)	17 (50.00%)	15 (53.57%)	1 (50.00%)	3 (50.00%)	
*Severely Obese*	8 (11.43%)	3 (8.82%)	3 (10.71%)	1 (50.00%)	1 (16.67%)	
*Morbidly Obese*	6 (8.57%)	2 (5.88%)	4 (14.29%)	0 (0.00%)	0 (0.00%)	
Chronic Disease History						0.074
*Diabetes mellitus*	5 (7.14%)	3 (8.82%)	2 (7.14%)	0 (0.00%)	0 (0.00%)	
*Hipertension*	25 (35.71%)	11 (32.35%)	7 (25.00%)	1 (50.00%)	6 (100.00%)	
*Diabetes mellitus + Hipertension*	15 (21.43%)	9 (26.47%)	5 (17.86%)	1 (50.00%)	0 (0.00%)	
*Other*	5 (7.14%)	4 (11.76%)	1 (3.57%)	0 (0.00%)	0 (0.00%)	
Surgical History	12 (17.14%)	5 (14.71%)	6 (21.43%)	0 (0.00%)	1 (16.67%)	0.821
Smoking	5 (7.14%)	3 (8.82%)	0 (0.00%)	1 (50.00%)	1 (16.67%)	0.034
HPV Status						0.978
*Negative*	56 (80.00%)	25 (96.15%)	26 (96.30%)	1 (100.00%)	4 (100.00%)	
*Positive*	2 (2.86%)	1 (3.85%)	1 (3.70%)	0 (0.00%)	0 (0.00%)	
Disease Stage						0.321
*Stage I*	17 (24.29%)	6 (17.65%)	9 (32.14%)	0 (0.00%)	2 (33.33%)	
*Stage II*	17 (24.29%)	5 (14.71%)	9 (32.14%)	1 (50.00%)	2 (33.33%)	
*Stage III*	23 (32.86%)	13 (38.24%)	8 (28.57%)	1 (50.00%)	1 (16.67%)	
*Stage IV*	13 (18.57%)	10 (29.41%)	2 (7.14%)	0 (0.00%)	1 (16.67%)	
Mortality						0.828
*Alive*	63 (90.00%)	30 (88.24%)	26 (92.86%)	2 (100.00%)	5 (83.33%)	
*Deceased*	7 (10.00%)	4 (11.76%)	2 (7.14%)	0 (0.00%)	1 (16.67%)	

**Table 2:** SUVmax values by Histopathological Type.

Histopathological Type	SUVmax (Mean ± SD)	*p*-Value
Serous Carcinoma	16.1 ± 10.4	0.336
Endometrioid Adenocarcinoma	14.8 ± 12.6	
Clear Cell Carcinoma	17.9 ± 10.2	
Carcinosarcoma	12.4 ± 2.9	

**Table 3:** SUVmax values by Survival Status.

Category	SUVmax (Mean ± SD)	*p*-Value
Overall (*n* = 70)	16.1 ± 10.4	0.376
Survival (*n* = 63)	16.6 ± 10.7	
Exitus (*n* = 7)	11.8 ± 6.9	

**Table 4:** SUVmax values by Distant Metastasis Status.

Category	SUVmax (Mean ± SD)	*p*-Value
Overall (*n* = 70)	16.1 ± 10.4	0.017
No distant metastasis (*n* = 63)	17.15 ± 10.4	
Distant metastasis (*n* = 7)	7.31 ± 4.4	

**Table 5:** Comparison of Preoperative Histopathological, Cytological, and Radiological Findings with Postoperative Histological Diagnoses in Endometrial Carcinoma Subtypes.

	Serous Carcinoma (*n* = 29)	Endometrioid Adenocarcinoma (*n* = 25)	Clear Cell Carcinoma (*n* = 2)	Mixed Cell Carcinoma (*n* = 6)	Mixed Cell Carcinoma (*n* = 8)	*p*-Value
**Endometrial Biopsy (Preoperative)**						<0.001
*Serous Carcinoma*	27 (93.10%)	5 (20.00%)	1 (50.00%)	0 (0.00%)	1 (12.50%)	
*Endometrioid Adenocarcinoma*	1 (3.45%)	19 (76.00%)	0 (0.00%)	4 (66.67%)	4 (50.00%)	
*Clear Cell Carcinoma*	0 (0.00%)	0 (0.00%)	1 (50.00%)	0 (0.00%)	1 (12.50%)	
*Mixed Cell Carcinoma*	1 (3.45%)	1 (4.00%)	0 (0.00%)	2 (33.33%)	2 (25.00%)	
**Smear Results**						0.640
*Benign Findings*	17 (70.83%)	19 (82.61%)	1 (100.00%)	3 (60.00%)	7 (100.00%)	
*Low-Grade Abnormalities*	3 (12.50%)	1 (4.35%)	0 (0.00%)	0 (0.00%)	0 (0.00%)	
*High-Grade Abnormalities*	3 (12.50%)	3 (13.04%)	0 (0.00%)	1 (20.00%)	0 (0.00%)	
*Malignancy*	1 (4.17%)	0 (0.00%)	0 (0.00%)	1 (20.00%)	0 (0.00%)	
**MRI Findings**						
*Mass Lesion*	9 (31.03%)	10 (40.00%)	1 (50.00%)	2 (33.33%)	5 (62.50%)	0.587
*Increased Endometrial Thickness*	5 (17.24%)	8 (32.00%)	0 (0.00%)	1 (16.67%)	0 (0.00%)	0.295
*Local Involvement*	22 (75.86%)	20 (80.00%)	2 (100.00%)	5 (83.33%)	8 (100.00%)	0.571
*Distant Metastasis*	4 (13.79%)	1 (4.00%)	0 (0.00%)	0 (0.00%)	0 (0.00%)	0.472
**PET/CT Findings**						
*Increased Involvement*	26 (89.66%)	24 (96.00%)	2 (100.00%)	6 (100.00%)	8 (100.00%)	0.688
*Increased Uptake*	4 (13.79%)	8 (32.00%)	2 (100.00%)	3 (50.00%)	3 (37.50%)	0.042
*Lesion*	2 (6.90%)	7 (28.00%)	0 (0.00%)	2 (33.33%)	3 (37.50%)	0.146
*Omental Cake*	3 (10.34%)	2 (8.00%)	0 (0.00%)	0 (0.00%)	0 (0.00%)	0.789
*Local Spread*	26 (89.66%)	24 (96.00%)	2 (100.00%)	6 (100.00%)	8 (100.00%)	0.688
*Distant Metastasis*	5 (17.24%)	2 (8.00%)	0 (0.00%)	0 (0.00%)	0 (0.00%)	0.466
**SUVmax**	14.8 ± 12.58	17.96 ± 10.18	18.65 ± 12.23	12.35 ± 2.92	16.95 ± 6.2	0.336

**Table 6:** Comparison of Preoperative and Postoperative Findings According to Mortality Outcomes.

	Alive (*n* = 63)	Deceased (*n* = 7)	*p*-Value
**Smear Results**			0.719
*Benign Findings*	43 (78.18%)	4 (80.00%)	
*Low-Grade Abnormalities*	4 (7.27%)	0 (0.00%)	
*High-Grade Abnormalities*	6 (10.91%)	1 (20.00%)	
*Malignancy*	2 (3.64%)	0 (0.00%)	
**Endometrial Biopsy (Preoperative)**			0.673
*Serous Carcinoma*	30 (47.62%)	4 (57.14%)	
*Endometrioid Adenocarcinoma*	26 (41.27%)	2 (28.57%)	
*Clear Cell Carcinoma*	2 (3.17%)	0 (0.00%)	
*Mixed Cell Carcinoma*	5 (7.94%)	1 (14.29%)	
**Histopathological Results (Postoperative)**			>0.999
*Serous Carcinoma*	26 (41.27%)	3 (42.86%)	
*Endometroid Adenocarcinoma*	22 (34.92%)	3 (42.86%)	
*Clear Cell Carcinoma*	2 (3.17%)	0 (0.00%)	
*Mixt Cell Carcinoma*	6 (9.52%)	0 (0.00%)	
*Carcinosarcoma*	7 (11.11%)	1 (14.29%)	
**MRI Findings**			
*Mass Lesion*	23 (36.51%)	4 (57.14%)	0.417
*Increased Endometrial Thickness*	13 (20.63%)	1 (14.29%)	>0.999
*Local Involvement*	50 (79.37%)	7 (100.00%)	0.334
*Distant Metastasis*	4 (6.35%)	1 (14.29%)	0.419
**PET/CT Findings**			
*Increased Involvement*	59 (93.65%)	7 (100.00%)	>0.999
*Increased Uptake*	19 (30.16%)	1 (14.29%)	0.664
*Lesion*	11 (17.46%)	3 (42.86%)	0.137
*Omental Cake*	5 (7.94%)	0 (0.00%)	>0.999
*Local Spread*	59 (93.65%)	7 (100.00%)	>0.999
*Distant Metastasis*	6 (9.52%)	1 (14.29%)	0.538
**SUVmax**	16.61 ± 10.67	11.83 ± 6.93	0.376

**Table 7:** Comparison of Clinical, Radiological, and Pathological Findings Across Cancer Stages.

	Stage I (*n* = 17)	Stage II (*n* = 17)	Stage III (*n* = 23)	Stage IV (*n* = 13)	*p*-Value
**Smear Results**					0.434
*Benign Findings*	10 (76.92%)	13 (92.86%)	17 (77.27%)	7 (63.64%)	
*Low-Grade Abnormalities*	1 (7.69%)	0 (0.00%)	2 (9.09%)	1 (9.09%)	
*High-Grade Abnormalities*	2 (15.38%)	1 (7.14%)	1 (4.55%)	3 (27.27%)	
*Malignancy*	0 (0.00%)	0 (0.00%)	2 (9.09%)	0 (0.00%)	
**Endometrial Biopsy (Preoperative)**					0.321
*Serous Carcinoma*	6 (35.29%)	5 (29.41%)	13 (56.52%)	10 (76.92%)	
*Endometrioid Adenocarcinoma*	9 (52.94%)	9 (52.94%)	8 (34.78%)	2 (15.38%)	
*Clear Cell Carcinoma*	0 (0.00%)	1 (5.88%)	1 (4.35%)	0 (0.00%)	
*Mixed Cell Carcinoma*	2 (11.76%)	2 (11.76%)	1 (4.35%)	1 (7.69%)	
**Histopathological Results (Postoperative)**					0.164
*Serous Carcinoma*	4 (23.53%)	4 (23.53%)	11 (47.83%)	10 (76.92%)	
*Endometroid Adenocarcinoma*	9 (52.94%)	7 (41.18%)	7 (30.43%)	2 (15.38%)	
*Clear Cell Carcinoma*	1 (5.88%)	1 (5.88%)	0 (0.00%)	0 (0.00%)	
*Mixt Cell Carcinoma*	1 (5.88%)	3 (17.65%)	1 (4.35%)	1 (7.69%)	
*Carcinosarcoma*	2 (11.76%)	2 (11.76%)	4 (17.39%)	0 (0.00%)	
**MRI Findings**					
*Mass Lesion*	7 (41.18%)	5 (29.41%)	11 (47.83%)	4 (30.77%)	0.611
*Increased Endometrial Thickness*	4 (23.53%)	4 (23.53%)	4 (17.39%)	2 (15.38%)	0.911
*Local Involvement*	15 (88.24%)	13 (76.47%)	19 (82.61%)	10 (76.92%)	0.803
*Distant Metastasis*	0 (0.00%)	0 (0.00%)	2 (8.70%)	3 (23.08%)	0.053
**PET/CT Findings**					
*Increased Involvement*	15 (88.24%)	16 (94.12%)	23 (100.00%)	12 (92.31%)	0.45
*Increased Uptake*	2 (11.76%)	6 (35.29%)	9 (39.13%)	3 (23.08%)	0.243
*Lesion*	3 (17.65%)	4 (23.53%)	5 (21.74%)	2 (15.38%)	0.939
*Omental Cake*	0 (0.00%)	0 (0.00%)	0 (0.00%)	5 (38.46%)	<0.001
*Local Spread*	15 (88.24%)	16 (94.12%)	23 (100.00%)	12 (92.31%)	0.45
*Distant Metastasis*	0 (0.00%)	0 (0.00%)	1 (4.35%)	6 (46.15%)	<0.001
**SUVmax**	13.79 ± 7.02	18.21 ± 10.61	17.69 ± 11.96	13.15 ± 10.43	0.349

**Table 8:** Association Between Smoking Status and Histopathological Subtypes.

Histopathological Types	No Smoking (*n* = 65)	Smoking (*n* = 5)	*p*-Value
Serous Carcinoma	27 (93.1%)	2 (6.9%)	0.019
Endometrioid Adenocarcinoma	25 (100.0%)	0 (0.0%)	
Clear Cell Carcinoma	1 (50.0%)	1 (50.0%)	
Mixed Cell Carcinoma	6 (75.0%)	2 (25.0%)	
Carcinosarcoma	6 (75.0%)	2 (25.0%)	

## Data Availability

The original contributions presented in this study are included in the article. Further inquiries can be directed to the corresponding author.
